# Preoperative Disc Angle is an Important Predictor of Segmental Lordosis After Degenerative Spondylolisthesis Fusion

**DOI:** 10.1177/21925682221118845

**Published:** 2022-08-10

**Authors:** Abdulmajeed Alahmari, Patrick Thornley, Andrew Glennie, Jennifer C. Urquhart, Fares Al-Jahdali, Raja Rampersaud, Charles Fisher, Fawaz Siddiqi, Parham Rasoulinejad, Christopher S. Bailey

**Affiliations:** 1 Division of Orthopaedics, Department of Surgery, 6221 Western University, London, ON, Canada; 2 Department of Orthopedics and Neurosurgery, 12361 Dalhousie University, Halifax, NS, Canada; 3 Lawson Health Research Institute, London, ON, Canada; 4 7938 University of Toronto, Toronto, Ontario, Canada; University Health Network, Toronto, Ontario, Canada; Arthritis Program, Krembil Research Institute, Toronto, Ontario, Canada; 5 University of British Columbia, Vancouver, British Columbia, Canada; Vancouver General Hospital, Vancouver, BC, Canada

**Keywords:** lumbar interbody fusion, posterolateral fusion, degenerative, degenerative spondylolisthesis, sagittal alignment, lumbar lordosis, sagittal balance

## Abstract

**Study Design:**

Retrospective Cohort Study

**Objectives:**

To determine the effect of interbody cages inserted via posterior approach on segmental lordosis in the setting of preoperative lordotic vs kyphotic discs in patients with lumbar degenerative spondylolisthesis (LDS).

**Methods:**

Retrospective analysis of prospectively collected data on assessment and management of LDS patients from 2 contributing centres. Patients were analyzed preoperatively and at 12-month follow-up with standing lumbar radiographs. Index level segmental lumbar lordosis (SLL), disc angle and global lumbar lordosis was measured. Patients were stratified into 4 groups based on index level disc angle and procedure: preoperative lordotic posterolateral fusion (group L-PLF); preoperative kyphotic PLF (group K-PLF); preoperative lordotic interbody fusion (IF) (group L-IF); preoperative kyphotic IF (group K-IF).

**Results:**

A total of 100/111 (90%) patients completed follow-up with 40 in group L-IF and 48 in group K-IF. There were 18 patients in group L-PLF and 5 in group K-PLF. Among patients with preoperatively lordotic disc angles who had a worsening of SLL, group L-IF had worse SLL than group L-PLF patients, with differences persisting at one-year (mean difference 2.30, 95% CI, .3, 4.3, *P* = .029). Patients in group K-IF achieved improvement in SLL at one-year more frequently than group L-IF (67% vs 44%, *P* = .046), with similar mean improvement magnitude between groups L-IF and K-IF (−1.1, 95% CI, −3.7, 1.6, *P* = .415).

**Conclusion:**

Segmental lordosis worsening was greater with preoperative index lordotic disc angles when an interbody cage was used. Patients who have a kyphotic disc preoperatively gain more lordosis with interbody cage use.

Patients with lumbar degenerative spondylolisthesis (LDS) and associated symptomatic spinal stenosis often experience significant functional improvement following surgical intervention.^1^ Interbody cages for fusion have not demonstrated significant clinical advantages when compared with posterolateral fusion.^2,3^ Despite these comparative investigations, interbody cages continue to be used widely. There are some potential advantages of interbody cages, however, calls for more selective usage of these expensive implants, in addition to accounting for patient-specific factors may lead to more responsible resource utilization for patients who truly require the technology. One of several potential advantages of interbody devices is to maintain or improve sagittal alignment which is important given that spinopelvic balance correlates with functional outcome following fusion for LDS.^4^

During lumbar surgery, maintaining global sagittal balance is dependent upon lumbar lordosis, the majority of which is dependent on the lower lumbar motion segments and specifically segmental disc angles.^5,6^ LDS can be associated with hypolordosis through the level of listhesis.^7^ Inadequate restoration of focal lordosis can lead to positive sagittal balance which is associated with increased risks of adjacent segment disease and worsened clinical outcomes as patients may continue to lean forward.^8-10^ It is important to understand how different types of single level spinal fusion affect lordosis in LDS to avoid a potential iatrogenic deformity by changing a previously lordotic disc to kyphotic or by failing to address a kyphotic disc.

In this investigation our objective was to determine the effect of interbody cages, inserted via a posterior approach, on segmental lordosis in the setting of index level preoperative lordotic vs kyphotic discs. We hypothesized that the effect of an interbody cage to increase segmental lordosis is larger and more pronounced when treating a kyphotic disc than when treating a lordotic disc. Furthermore, we hypothesized that an interbody cage could have a detrimental effect on segmental lordosis in the setting of a preoperative lordotic disc.

## Methods

### Patients and Clinical Data

A retrospective analysis of prospectively collected data between January 12 015 and December 31, 2020, from 2 contributing Canadian Spine Outcomes Research Network (CSORN) centres on the assessment and management of lumbar degenerative spondylolisthesis patients. Patients were included if they met the following inclusion criteria: radiographic evidence of degenerative spondylolisthesis with symptoms of neurogenic claudication or radiculopathy with or without back pain, unresponsive to non-operative management over at least 3 months and underwent surgical treatment between January 1, 2015 and Feb 28, 2020 at 2 tertiary care academic spine centers by either posterolateral fusion or interbody fusion for 1 lumbar spine segment. Patients who had multilevel decompressions for spinal stenosis in the same procedure were included if the instrumented fusion was limited to 1 segment. Patients with greater than 10 degrees of scoliosis were excluded. Patients who underwent surgery for isthmic spondylolisthesis, spinal fracture, concomitant cervical or thoracic myelopathy, multilevel fusion procedures or had previous lumbar fusion procedures were excluded. Additionally, patients with concomitant hip and/or knee osteoarthritis were excluded from the analysis. All patients provided written consent to participate in the study. Study approval was provided by the Western University Health Science Research Ethics Board, approval number 103079.

### Patient Characteristics, Operative Details and Radiological Measurements

Standardized CSORN procedure, questionnaire and data collection forms were utilized amongst both contributing centres. Patient demographics captured included age, body mass index (BMI), sex, comorbidities and smoking history. Diagnosis data including primary complaint, sensory or motor deficit and grade of spondylolisthesis were reported by the surgeon at the preoperative initial consultation. Operative data including the type of procedure (posterolateral vs interbody fusion), operating time, blood loss, and intraoperative adverse events were collected.

While the expressed goal of each surgical intervention was not to primarily maximize lumbar lordosis, a standardized approach was utilized among all participating surgeons to enhance postoperative lumbar lordosis among all included patients. All operative procedures were performed by fellowship trained spine surgeons. Each patient was positioned prone on the Jackson frame spine table, with an open midline posterior approach performed. To enhance lordosis, each decompression performed included a complete bilateral medial facetectomy (inferior articular process resection). Interbody cages utilized were all non-expandable and were either Medtronic Capstone™ or Fuse™ cages. Interbody cages were inserted as anterior within the disc space as possible with symmetric bilateral compression performed across both sided pedicle screw-rod constructs.

Post-operative length of stay and adverse events were collected at discharge. Adverse events were also reported at routine follow-up visits at 6-18 weeks and 1-year after surgery. Prior to surgery and at routine follow-up visits (6-18 weeks and 1-year) patients had standing and recumbent lumbar lateral radiographs and a standing 36-inch lateral radiograph of the entire spine including the femoral heads. Sacral slope (SS), pelvic incidence (PI), sagittal vertical axis (SVA), pelvic tilt (PT), thoracic kyphosis (TK), T1 spinopelvic inclination and T9 spinopelvic inclination were measured according to the previously described methodology of Radanovic et al.^4^ Slip percentage, disc angle (lordotic or kyphotic), were measured by the treating surgeon. On the standing lumbar lateral radiographs global lumbar lordosis was measured from the upper endplate of L1 to the upper end plate of S1. Focal lumbar lordosis was measured from the upper end plate of the proximal vertebra to lower end plate of distal vertebra at the index surgical level preoperatively.^11,12^

### Statistical Analysis

Data were analyzed using SPSS software version 26 (SPSS Inc., Chicago, IL, USA). Patients were subdivided into 4 groups according to preoperative standing disc angle (lordotic or kyphotic) and type of fusion (posterolateral or interbody). For continuous parametric variables between group comparisons were made using a one-way analysis of variance (ANOVA) with a least squares difference post hoc test. The Kruskal-Wallis test was used for all continuous non-parametric variables. Comparisons of categorical variables were made using the chi-square test or Fisher exact test.

For focal lumbar lordosis the change in score was calculated by subtracting the preoperative baseline value from the 6–18 week or 1-year value. An increase in lordosis was denoted by a positive change in score whereas a decrease in lordosis was denoted by a negative change in score. A Student’s t-test was used to compare focal lumbar lordosis according to fusion type in patients with a pre-operative lordotic disc angle and to compare focal lumbar lordosis according to preoperative disc angle in patients with an interbody fusion. A sensitivity analysis was performed with age, slip (mm), foraminal height and site as covariates. A *P*-value of less than .05 was considered to indicate statistical significance.

## Results

A total of 111 patients were eligible for inclusion in our analysis. Twenty-three patients (22%) received posterolateral fusion (PLF) surgery of whom 18 had a lordotic preoperative disc angle (Group L-PLF) while only 5 a had kyphotic preoperative disc angle (Group K-PLF). Eighty-eight patients (79%) underwent interbody fusion (IF) including 40 patients that had a lordotic preoperative disc angle (Group L-IF) and 48 patients that had a kyphotic preoperative disc angle (Group K-IF). Out of 111 patients included, 100 patients had radiographic data available at the 1-year postoperative timepoint.

### Baseline Characteristics

The baseline characteristics and demographics are shown in Table 1. Patients that had a posterolateral fusion were older (69.6 ± 6.6 years (group L-PLF) and 72.6 ± 6.8 years (group K-PLF)) than those that had an interbody fusion and a kyphotic disc (63.7 ± 9.3 years (group K-IF); *P* < .05). Patients that had a posterolateral fusion were more likely to have preoperative sensory and/or motor deficits (44%, group L-PLF and 40%, group K-PLF) as opposed to patients that had an interbody fusion (18%, group L-IF and 13%, group K-IF; *P* = .023). Otherwise, the groups were similar in that the majority of patients in each group were female and had an average BMI of 26 to 32 kg/m^2^, a grade 1 spondylolisthesis, a primary complaint of neurogenic claudication and an average of 3 comorbidities. There were 4 patients (8.3%) who had previous decompressive microdiscectomy surgery in group K-IF and 1 patient in group L-PLF; *P* = .283.

**Table 1.** Preoperative Patient Characteristics.

Parameter	Pre-op Lordotic, Posterolateral Fusion (Group L-PLF) n = 18	Pre-op Kyphotic, Posterolateral Fusion (Group K-PLF) n = 5	Pre-op Lordotic, Interbody Fusion (Group L-IF) n = 40	Pre-op Kyphotic, Interbody Fusion (Group K-IF) n = 48	*P*-value
Age, years					
Mean±SD	69.6 ± 6.6^a^	72.6 ± 6.8^a^	66.8 ± 7.9	63.7 ± 9.3	.021
Body mass index, kg/m^2^					
No. of patients	17	5	40	47	.257
Mean±SD	29.5 ± 4.0	26.2 ± 6.2	31.1 ± 7.6	31.7 ± 6.3	
Sex, Female, n (%)	11 (61.1)	5 (100.0)	26 (65.0)	39 (81.3)	.107
Current smoker, n (%)	2 (11.1)	0 (.0)	7 (17.5)	8 (17.0)	.708
Primary Complaint, n (%)					
Back pain	0 (.0)	0 (.0)	0 (.0)	1 (2.1)	.697
Neurogenic claudication	15 (83.3)	4 (80.0)	37 (92.5)	44 (91.7)	
Radiculopathy	3 (16.7)	1 (20.0)	3 (7.5)	3 (6.3)	
Sensory or Motor Deficit, n (%)	8 (44.4)	2 (40.0)	7 (17.5)	6 (12.5)	.023
Grade Spondylolisthesis, n (%)					
Grade 1	14 (77.8)	3 (60.0)	31 (75.0)	30 (64.6)	.608
Grade 2	3 (16.7)	2 (40.0)	10 (25.0)	16 (33.3)	
Grade 3	1 (5.6)	0 (.0)	0 (.0)	1 (2.1)	
Previous Surgery, n (%)	1 (5.6)	0 (.0)	0 (.0)	4 (8.3)	.283
Number of Comorbidities, n (%)^b^					
No. of patients	18	5	40	46	.284
Mean±SD	3.5 ± 2.4	4.6 ± 2.5	3.3 ± 1.4	3.9 ± 2.0	

^a^P < .05 vs kyphotic interbody.

^b^Patients could have more than 1 comorbidity.

### Operative Details and Postoperative Adverse Events

Procedural details are shown in Table 2. All patients had a single level instrumented fusion occurring most commonly at the L4-5 level. There was no significant difference between groups with regard to operating time (*P* = .347), blood loss (*P* = .641), length of stay (*P* = .211) or intraoperative adverse events (*P* > .05 all comparisons). Eighty-nine percent of group L-PLF and 100% of group K-PLF patients had an ASA score of ≧3 whereas 43% and 31% of patients in groups L-IF and K-IF respectively had a score of ≧3 (*P* = .001). The groups differed in surgical approach in that all patients in groups L-PLF and K-PLF had an open approach whereas 11 patients (27.5%) in group L-IF and 3 patients (6.3%) in group K-IF had a minimally invasive approach (*P* = .004). Perioperative and postoperative adverse events did not differ between any of the groups (Table 3).

**Table 2.** Operative Details.

Parameter	Pre-op Lordotic, Posterolaeral Fusion (Group L-PLF) n = 18	Pre-op Kyphotic, Posterolateral Fusion (Group K-PLF) n = 5	Pre-op Lordotic, Interbody Fusion (Group L-IF) n = 40	Pre-op Kyphotic, Interbody Fusion (Group K-IF) n = 48	*P*-Value
Operated levels, n (%)^a^					.394
1	17 (94.4)	5 (100.0)	38 (95.0)	46 (95.8)	
2	0 (.0)	0 (.0)	2 (5.0)	2 (4.2)	
3	1 (5.6)	0 (.0)	0 (.0)	0 (.0)	
Level Fused, n (%)					.347
L3/L4	4 (22.2)	0 (.0)	3 (7.5)	6 (12.5)	
L4/L5	14 (77.8)	5 (100.0)	37 (92.5)	42 (87.5)	
Operating time (min)					.356
No. of Patients	18	5	40	47	
Mean±SD	150 ± 38	160 ± 37	171 ± 45	152 ± 37	
Median (min – max)	147 (91–238)	151 (119–202)	158 (118–275)	150 (78–238)	
Blood loss (min)					.641
No. of Patients	14	5	32	47	
Mean ± SD	455 ± 225	490 ± 152	472 ± 272	396 ± 147	
Median (min – max)	400 (225–1000)	600 (300–600)	400 (150–1400)	400 (100–800)	
Length of stay (days)					.211
Mean ± SD	4.0 ± 2.0	4.4 ± 1.5	3.2 ± 1.3	3.7 ± 2.5	
Median (min – max)	3 (2–10)	4 (3–6)	3 (1–7)	3 (1–13)	
ASA score					.001
2	2 (11.1)	0 (.0)	23 (57.5)	33 (68.8)	
3	16 (88.9)	5 (100.0)	16 (40.0)	13 (27.1)	
4	0 (.0)	0 (.0)	1 (2.5)	2 (4.2)	
Intraoperative adverse events, n (%)^b^					
No. with at least 1 AE	5 (27.8)	2 (40.0)	4 (10.0)	8 (16.7)	.193
Dural tear	4 (22.2)	1 (20.0)	3 (7.5)	3 (6.3)	.203
Implant instrument Related	0 (.0)	0 (.0)	0 (.0)	2 (4.2)	.445
Revision intra-operative	1 (5.6)	1 (20.0)	2 (5.0)	3 (6.3)	.633
Minimally invasive approach, n (%)	0 (.0)	0 (.0)	11 (27.5)	3 (6.3)	.004

AE, adverse event; ASA, American Society of Anesthesiologists Score; One-way ANOVA Age, BMI = LSD post-hoc comparison if used Bonferroni, no significant difference between age groups. Kruskal-Wallis Test operating time, blood loss, length of stay.

^a^Includes fused level and levels decompressed.

^b^Patients could have more than 1 adverse event.

**Table 3.** Perioperative and Postoperative Complications.^a^

Parameter	Pre-op Lordotic, Posterolateral Fusion (Group L-PLF) n = 18	Pre-op Kyphotic, Posterolateral Fusion (Group K-PLF) n = 5	Pre-op Lordotic, Interbody Fusion (Group L-IF) n = 40	Pre-op Kyphotic, Interbody Fusion (Group K-IF) n = 48	*P*-.alue
Perioperative adverse event, n (%)					
No. patients with at least 1 AE	3 (16.7)	0 (.0)	3 (7.5)	8 (16.7)	.448
Delirium	0 (.0)	0 (.0)	0 (.0)	1 (2.1)	.723
Fall	0 (.0)	0 (.0)	0 (.0)	1 (2.1)	.723
Gastrointestinal bleeding	1 (5.6)	0 (.0)	0 (.0)	0 (.0)	.157
Ileus	0 (.0)	0 (.0)	0 (.0)	1 (2.1)	.723
Deep wound infection	0 (.0)	0 (.0)	0 (.0)	1 (2.1)	.723
New onset pain	0 (.0)	0 (.0)	1 (2.5)	2 (4.2)	.792
Urinary retention	0 (.0)	0 (.0)	0 (.0)	1 (2.1)	.723
Other	3 (16.7)	0 (.0)	2 (5.0)	2 (4.2)	.252
Post-op AE <18 weeks, n (%)					
No. patients with at least 1 AE	3 (18.8)	0 (.0)	7 (17.5)	12 (25.0)	.514
Implant instrument related	0 (.0)	0 (.0)	1 (2.5)	1 (2.1)	.907
Deep wound infection	0 (.0)	0 (.0)	1 (2.5)	5 (10.4)	.228
Superficial infection	0 (.0)	0 (.0)	2 (5.0)	1 (2.1)	.697
Urinary tract infection	1 (5.6)	0 (.0)	0 (.0)	0 (.0)	.157
Neurological deterioration	0 (.0)	0 (.0)	1 (2.5)	0 (.0)	.617
Pain new onset	0 (.0)	0 (.0)	1 (2.5)	2 (4.2)	.792
Urinary retention	1 (5.6)	0 (.0)	0 (.0)	0 (.0)	.157
Wound dehiscence	0 (.0)	0 (.0)	1 (2.5)	1 (2.1)	.907
Serous wound drainage	1 (5.6)	0 (.0)	0 (.0)	0 (.0)	.157
Other	0 (.0)	0 (.0)	2 (5.0)	1 (2.1)	.697
Post-op >18 weeks AE, n (%)					
No. patients with at least 1 AE	1 (5.6)	0 (.0)	1 (2.5)	7 (14.5)	.172
Deep wound infection	0 (.0)	0 (.0)	(.0)	3 (6.3)	.256
Non-union	0 (.0)	0 (.0)	0 (.0)	2 (4.2)	.445
Adjacent segment degeneration	0 (.0)	0 (.0)	1 (2.5)	1 (2.1)	.909
Other	1 (5.6)	0 (.0)	2 (2.5)	2 (4.2)	.956
Second surgeries, n (%)	0 (.0)	0 (.0)	4 (10.0)	11 (22.9)	.054
Third surgeries, n (%)	0 (.0)	0 (.0)	0 (.0)	3 (6.3)	.256

^a^Patients could have more than 1 adverse event.

### Preoperative Radiographic Measures

In patients that had a lordotic preoperative disc angle, the average was largest in group L-PLF, 7.7 ± 3.7°, compared to group L-IF, 5.3 ± 3.1° (*P* = .001) (Table 4). Similarly, for patients with a preoperative kyphotic disc angle there was a statistically significant difference between group K-PLF, −3.1 ± 1.8°, and group K-IF, -2.0 ± 3.9° (*P* = .001). When the sagittal vertical axis (SVA) was compared to group K-IF, 23.6 ± 43.0mm, the SVA was greater in patients undergoing posterolateral fusion regardless of disc type, 46.9 ± 32.1 mm, group L-PLF and 77.4 ± 30.4mm, K-PLF. The T1 spinal pelvic incidence (T1SPI) was smaller in group L-PLF vs group K-IF (1.6 ± 2.5°, group L-PLF vs 4.4 ± 4.4°, group K-IF, *P* = .029).

**Table 4.** Average Preoperative Radiographic Measures.

Parameter	Pre-op Lordotic, Posterolateral Fusion (Group L-PLF)			Pre-op Kyphotic, Posterolateral Fusion (Group K-PLF)			Pre-op Lordotic, Interbody Fusion (Group L-IF)			Pre-op Kyphotic, Interbody Fusion (Group K-IF)			*P*-value
	n	Mean	SD	n	Mean	SD	n	Mean	SD	n	Mean	SD	
Slip Percentage (%)	18	20.7	12.2	5	25.3	9.5	40	20.1	6.9	48	29.3	33.7	.278
Disc Angle, (°)	18	7.7^abd^	3.7	5	-3.1^bc^	1.8	40	5.3^acd^	3.1	48	-2.0^bc^	3.9	.001
SVA, (°)	16	46.9^a^	32.1	4	77.4^ab^	30.4	38	27.9	32.1	48	23.6	43.0	.015
SS, (°)	17	34.5	9.3	5	31.2	10.9	40	36.0	9.2	48	31.2	7.7	.067
PT, (°)	17	23.4	6.8	5	30.5	4.6	40	22.8	8.3	48	25.1	9.7	.232
PI, (°)	17	58.1	12.5	5	62.3	15.8	40	58.0	13.4	48	55.5	12.3	.603
TK, (°)	16	37.5	12.6	4	44.0	13.7	39	38.5	11.9	47	35.8	12.8	.529
T1 SPI, (°)	16	1.6#	2.5	4	1.5	3.8	35	2.9	2.4	47	4.4	4.4	.029
T9 SPI, (°)	16	8.8	2.9	4	11.8	5.0	37	9.9	3.3	47	8.8	4.8	.330
Foraminal Ht, mm	18	19.7	2.5	5	21.6	2.5	32	18.7	2.5	35	18.1	4.3	.093

^a^*P* < .05 vs Interbody kyphotic/neutral.

^b^*P* < .05 vs Interbody Lordotic.

^c^*P* < .05 vs PLF Lordotic.

^d^*P* < .05 vs PLF kyphotic/neutral.

### Comparison of Post-Operative Focal Lumbar Lordosis and Global Lumbar Lordosis

Patients that had a lordotic preoperative disc angle (groups L-PLF and L-IF) had a higher focal lumbar lordosis at baseline, 6–18 weeks, and 1-year compared to patients that had an interbody fusion and a kyphotic preoperative disc angle (group K-IF). At baseline, global lumbar lordosis was also greater in patients in groups L-PLF and L-IF vs group K-IF. Overall, the focal lumbar lordosis and global lumbar lordosis were not significantly changed except in group K-IF at 6–18 weeks, focal lumbar lordosis was improved by a mean difference of 3.3 ± 5.8°(*P* = .036), but the improvement was not statistically significant at 1-year measuring 2.4. ± 5.0° (*P* = .139). The numeric data for each group with mean difference at 6–18 weeks and 1-year are outlined in Table 5.

**Table 5.** Average and Average Change from Baseline in Focal and Global Lumbar Lordosis.

Parameter		Pre-op Lordotic, Posterolateral Fusion (Group L-PLF)			Pre-op Kyphotic, Posterolateral Fusion (Group K-PLF)			Pre-op Lordotic, Interbody Fusion (Group L-IF)			Pre-op Kyphotic, Interbody Fusion (Group K-IF)			P-Value
		n	Mean	SD	n	Mean	SD	n	Mean	SD	n	Mean	SD	
Focal lumbar lordosis	Baseline	18	16.7^a^	6.4	5	10.8	3.0	40	19.3^a^	6.4	48	10.4	8.8	.001
	6–18 wk	18	18.6^a^	6.8	5	11.2	1.7	39	19.1^a^	7.0	48	13.6	6.6	.001
	1 year	15	16.9^a^	6.1	4	11.9	3.1	39	19.1^a^	7.7	42	12.5	7.2	.001
	Δ6–18 wk	18	1.9	2.9	5	0.4	3.2	39	.03^a^	5.4	48	3.2	5.8	.036
	Δ12 mo	15	1.5	3.2	4	1.3	0.8	39	-0.1	5.4	42	2.4	5.0	.139
Global lumbar lordosis	Baseline	18	47.9^a^	12.3	5	42.3	17.1	39	47.1#	11.5	48	38.9	10.8	.004
	6–18 wk	18	50.2	9.8	5	48.2	8.5	38	50.2	14.4	46	46.2	9.9	.409
	1 year	14	49.0	11.8	4	42.3	10.1	37	52.0	12.5	36	46.8	11.9	.199
	Δ6–18 wk	18	2.3	7.8	5	5.9	8.9	38	4.3	11.9	46	7.3	8.6	.276
	Δ1 year	14	2.5	8.2	4	4.3	9.0	36	6.3	9.6	36	8.4	8.8	.211

Mean values are derived from one-way ANOVA using all available data. Change values are derived from one-way ANOVA using complete case analysis. Absolute change scores may differ from that derived from one-way ANOVA due to missing data.

Δ = change from baseline.

^a^*P* < .05 vs Interbody kyphotic/neutral

Comparison of change in focal lumbar lordosis according to fusion type in patients with a preoperative lordotic disc angle

While not statistically significantly different, at 6–18 weeks and 1-year postoperatively, a similar proportion of patients with a lordotic preoperative disc angle, groups L-PLF and L-IF, achieved improvement in focal lumbar lordosis (group L-PLF vs group L-IF; 6–18 weeks, 61.1% vs 46.2%; *P* = .395 and 1-year, 60% vs 44 %, *P* = .366; Table 6). The mean magnitude of this improvement was also similar between groups (group L-PLF vs group L-IF; 6–18 weeks, *P* = .395 and 1-year, *P* = .366; Table 6). However, those in group L-IF that had worsening in focal lordosis worsened by a significantly larger magnitude than those that had a posterolateral fusion (group L-PLF) at 6–18 weeks (mean difference, 3.0°. 95% CI, 1.1°, 4.9°, P=.004;) and this effect persisted at 1-year (mean difference, 2.3, 95% CI, .3, 4.3, *P* = .029; Table 6). This discrepancy in mean difference can be explained by the relatively small change in focal lumbar lordosis between preoperative and postoperative measurements for group L-PLF (−.9° ± .9° at 6–18 weeks; −1.4° ± 1.8° at 1 year) compared to group L-IF (−3.9° ± 2.4° at 6–18 weeks; −3.7° ± 2.3° at 1 year). A sensitivity analysis adjusting for baseline variables including age, slip (mm), foraminal height and hospital site confirmed similar significant magnitude in worsening in focal lordosis in the interbody fusion group.

**Table 6.** The Proportion of Patients that Had Improvement or Worsening/No Change in Focal Lumbar Lordosis and the Magnitude of Improvement or Worsening at 6-18 Weeks and 1-Year After Surgery According to Fusion Type in Patients With a Preoperative Lordotic Disc Angle.

	Pre-op Lordotic, Posterolateral Fusion (Group L-PLF) n = 18	Pre-op Lordotic, Interbody Fusion (Group L-IF) n = 40	Mean difference (95% CI)	*P*-Value
Focal Lumbar Lordosis				
Δ6-18 wk:				
% Worse/Same, n (%)	7 (38.9 %)	21 (53.8%)	—	.395
% Improved, n (%)	11 (61.1%)	18 (46.2%)		
Worse/Same, Mean ± SD	−.9 ± 0.9	−3.9 ± 2.4	3.0 (1.1, 4.9)	.004
Improved, Mean ± SD	3.7 ± 2.2	4.6 ± 4.3	−.9 (−3.8, 2.0)	.520
Δ1 year:				
% Worse/Same, n (%)	6 (40.0)	22 (56.4)	—	.366
% Improved, n (%)	9 (60.0)	17 (43.6)		
Worse/Same, Mean ± SD	−1.4 ± 1.8	−3.7 ± 2.3	2.3 (.3, 4.3)	.029
Improved, Mean ± SD	3.3 ± 2.5	4.5 ± 4.5	−1.2 (−4.6, 2.3)	.494

A positive change in score from baseline indicates an improvement in focal lumbar lordosis. Worsening focal lumbar lordosis is denoted by a negative change in score from baseline or no change (same).

### Comparison in Focal Lumbar Lordosis According to Preoperative Disc Angle in Patients with an Interbody Fusion

The percentage of patients whose disc angle was made better or worse after interbody fusion is shown in Table 7. Patients in group K-IF compared to group L-IF were more likely to achieve improvement in focal lumbar lordosis at 6-18 weeks (73% vs 46%, *P* = .015) and 1-year (67% vs 44%, *P* = .046); however, the mean magnitude of improvement was similar between the groups (group L-IF vs group K-IF, -1.1°, 95% CI, -3.7°, 1.6°, *P* = .415). A sensitivity analysis with adjustment for baseline variables confirmed a similar amount of improvement.

**Table 7.** The Proportion of Patients that Had Improvement or Worsening/No Change in Focal Lumbar Lordosis and the Magnitude of Improvement or Worsening at 6-18 Weeks and 1-Year After Surgery According to Preoperative Disc Angle in Patients with an Interbody Fusion.

	Pre-op Lordotic, Interbody Fusion (Group L-IF) n = 40	Pre-op Kyphotic, Interbody fusion (Group K-IF) n = 48	Mean Difference (95% CI)	P-Value
Focal lumbar lordosis				
Δ6-18 wk				
% Worse/Same, n (%)	21 (53.8)	13 (27.1)	—	.015
% Improved, n (%)	18 (46.2)	35 (72.9)		
Worse/Same, Mean ± SD	−3.9 ± 2.4	−3.2 ± 3.0	−.7 (−2.6, 1.2)	.470
Improved, Mean ± SD	4.6 ± 4.3	5.6 ± 4.6	−1.0 (−3.7, 1.6)	.415
Δ1 year				
% Worse/Same, n (%)	22 (56.4)	14 (33.3)	—	.046
% Improved, n (%)	17 (43.6)	28 (66.7)		
Worse/Same, Mean ± SD	−3.7 ± 2.3	−3.1 ± 2.9	−.6 (−2.3, 1.1)	.473
Improved, Mean ± SD	4.5 ± 4.6	5.2 ± 3.3	−.7 (−3.1, 1.7)	.558

A positive change in score from baseline indicates an improvement in focal lumbar lordosis. Worsening focal lumbar lordosis is denoted by a negative change in score from baseline or no change (same).

## Discussion

This study is part of an ongoing large multi-centered prospectively enrolled cohort of patients undergoing posterior fusion surgery with or without interbody cages for lumbar spondylolisthesis with comprehensive radiographic data. This investigation presents results from a sub-analysis of 2 of the participating centres, evaluating 111 patients with degenerative spondylolisthesis according to surgery type (posterolateral or interbody fusion) and preoperative disc angle (kyphotic vs lordotic). Our study shows that instrumented fusion, regardless of whether an interbody cage was used or not, had an insignificant effect on post-operative segmental lordosis, which may be related to an averaging effect between patients who improved and worsened. For this reason, we performed a sub-analysis separating patients that achieved post-operative improvement in lordosis from those that did not within each group. Our findings demonstrate that for patients with a pre-operative lordotic disc angle, although the proportion and magnitude of post-operative improvement were not significantly different between interbody fusion and posterolateral fusion groups, for those that actually lost lordosis post-operatively, the magnitude of worsening was greater when an interbody cage was used. Furthermore, our findings also suggest that patients who have a preoperative kyphotic disc are more likely to gain lordosis when an interbody cage is used when compared to posterolateral fusion alone. This suggests that interbody fusion should be strongly considered to address lumbar lordosis if the disc angle preoperatively is kyphotic, as that will have the greatest likelihood of desired impact without the significant risk of creating a flattening of the segmental lordosis as occurred with interbody cage use in preoperatively lordotic discs.

There remains clinical equipoise about the best technique and procedure type for patients with lumbar degenerative spondylolisthesis and it is known that surgeon training, belief and resource access largely dictate the type of most-preferred intervention.^13^ It is therefore important to apply a more precise, selective approach, accounting for patient, radiographic and surgical factors when determining the optimal procedure type for a given patient. One such factor may be the preservation or restoration of segmental lordosis which has gained increasing attention because of its relation to adjacent segment disease, global sagittal balance and poor postoperative outcomes.^4,14,15^ Studies looking at improvement of segmental lordosis following posterior interbody fusion procedures have shown conflicting results with some studies demonstrating improvement in segmental and global lumbar lordosis^16-19^, with other investigations showing a lack of significant improvement and even the potential for creating focal kyphosis.^20-22^ The optimal patient selection (eg risk of subsidence) for interbody fusion in addition to posterolateral instrumentation as well as differences in surgical goals, techniques and implant choices may explain in part the conflict in radiological outcome of interbody fusion in the literature.

One explanation of our study findings is that kyphotic discs had more correction potential to lordosis, making a positive lordotic change easier to achieve as compared to lordotic discs in which maintaining the lordosis may be more important than further gain. From what is known about the positive effect of prone positioning on lumbar and segmental lordosis^23^, 1 can expect posterolateral fusion to have less negative effects on an already lordotic segment which is what we showed in this study.

This study confirms similar findings on the effect of preoperative lordosis as an important factor to predict lordosis gain as previously shown by Berlin et al, which looked at a cohort of L4/L5 transforaminal lumbar interbody fusion (TLIF) patients.^24^ The authors found that a preoperative segmental lordosis cut off of more than 23° was a predictor of negative correction. However, it is not clear that all included patients in Berlin’s study had a diagnosis of degenerative spondylolisthesis potentially including lytic spondylolisthesis and other primary diagnoses at the L4/L5 referenced index level. Inculet et al have previously shown that the indication for interbody use is widely different between degenerative and lytic spondylolisthesis patient populations.^25^ We believe that the preoperative disc angle is a more direct parameter to look at before surgery rather than a cut off value of segmental lordosis because preoperative segmental lordosis can be affected by the vertebral body shape, which is frequently trapezoidal and may produce lordosis even though the disc might be kyphotic. Martin et al, analyzed factors that were associated with more than 5 degrees of lordosis gain in a cohort of patients who underwent TLIF and the authors found that kyphosis at the disc and gas phenomena had a significant positive correlation.^11^ Recently, Liu et al published results of 137 patients who underwent a TLIF procedure for isthmic and degenerative spondylolisthesis.^12^ In their review, a logistic regression analysis showed that disc angle and segmental lordosis were highly correlated with postoperative lordosis change which aligns with our findings at 6-18 weeks post-operatively but in our cohort the difference deceased over time which could be related to subsidence.^12^ However, we recognize the importance of considering other unrelated factors when determining indications for cage use in degenerative lumbar spondylolisthesis such as stability, fusion (in particular for minimally invasive techniques where an interbody is used in all cases regardless), and foraminal stenosis for example.

This study does have some limitations. Firstly, the included analysis is the result of a question posed retrospectively using patient and radiographic data that was collected prospectively in the CSORN database with the intent of evaluating the assessment and management of LDS patients. Secondly, the number of patients in the preoperative kyphotic posterolateral fusion group was small with only 5 available patients. This may be explained by surgeon bias to use an interbody cage in patients presenting with a kyphotic index level disc. Thirdly, it must be recognized that these single level fusion surgeries were largely performed to treat neurogenic claudication and it is possible that a goal of the operating surgeons was not deformity correction which could limit the degree of correction in some patients. Furthermore, other factors that were not specifically examined could affect the focal and global lumbar lordosis including differences in technique and interbody cage size, design and geometry as well as the extent of anterior placement of the cage.^19,22^ It should be noted that this study looked at interbody fusion from a more generalizable approach as it is performed in a real world every day scenario and makes no claim that this is the maximum correction in lordosis that can be achieved, a point that was also argued in previous studies.^11,12^ Furthermore, our study was purely radiographically focused and we did not correlate our findings with functional outcomes.

## Conclusion

This study contributes to the growing focus on sagittal alignment parameters for degenerative lumbar spondylolisthesis surgery and adds to the comparison and indication rationale for posterolateral fusion and interbody fusion based on preoperative disc angle. In the setting of a preoperatively lordotic disc angle, the use of a cage is not supported by our work as the magnitude of worsening in segmental lordosis is larger with interbody fusion than posterolateral fusion. We have also demonstrated that the global and segmental lumbar lordosis gain is more significant when interbody fusion is utilized in a patient with a preoperatively kyphotic index disc angle rather than a lordotic disc angle.
